# Retracted: DNA Binding and Photocleavage Studies of Cobalt(III) Polypyridine Complexes: [Co(en)_2_PIP]^3+^, [Co(en)_2_IP]^3+^, and [Co(en)_2_phen-dione]^3+^

**DOI:** 10.1155/2021/7161643

**Published:** 2021-01-19

**Authors:** Bioinorganic Chemistry and Applications

*Bioinorganic Chemistry and Applications* has retracted the article titled “DNA Binding and Photocleavage Studies of Cobalt(III) Polypyridine Complexes: [Co(en)_2_PIP]^3+^, [Co(en)_2_IP]^3+^, and [Co(en)_2_phen-dione]^3+^” [1].

This article has been retracted as it is essentially identical in title and technical content with a previously published paper in Polyhedron. The earlier publication is “DNA binding and cleavage properties of certain ethylenediamin cobalt(III) of modified 1,10-phenanthrolines,” Penumaka Nagababu and S. Satyanarayana, Polyhedron, Volume 26, Issue 8, Pages 1686–1692.
